# Enhancement of Rotenone Cytotoxicity in the Presence of Bach1 Inhibitors

**DOI:** 10.1134/S1607672925601817

**Published:** 2026-03-30

**Authors:** S. V. Nikulin, D. Olkhovik, A. V. Razumovskaya, M. O. Silkina, A. A. Zakhariants, K. V. Klycheva, G. A. Khairetdinova, I. G. Gazaryan, D. M. Hushpulian

**Affiliations:** 1 National Research University Higher School of Economics (HSE University), Moscow, Russia; 2 Shemyakin–Ovchinnikov Institute of Bioorganic Chemistry, Russian Academy of Sciences, Moscow, Russia; 3 Pirogov Russian National Research Medical University, Moscow, Russia; 4 Moscow State University, Moscow, Russia

**Keywords:** prostate cancer, colorectal cancer, Bach1, HPPE, rotenone, heme oxygenase 1

## Abstract

A novel trend in anticancer therapy is based on the combination of cytotoxic and metabolic drugs. Bach1 is a transcription factor activating glycolysis and increasing proliferation and metastatic potential in cancer cells. The cytotoxic effect was evaluated for a combination of rotenone, an inhibitor of mitochondrial respiration, and two different inhibitors of Bach1 transcription factor in the prostate cancer cell lines (PC3 and Du145) and colorectal cancer (HT-29 and HCT-116) in a kinetic mode. An enhancement of the cytotoxic action of the combination therapy was observed only for the prostate cancer cell lines PC3 and Du145. No enhancement of cytotoxicity of zinc porphyrin in the presence of rotenone was observed for the НСТ-116 line. The HT-29 line was not sensitive to either inhibitor or their combination with rotenone at 24 h incubation. According to the PCR results, HT-29 was the only line showing an extremely high activation of heme oxygenase 1 (HMOX1) in the presence of the Bach1 inhibitor, pointing to the highest level of the antioxidant defense in this cell line. The sensitivity of the prostate cancer cell lines to the combination therapy points to the significant differences in the metabolism between the prostate and colorectal cell lines.

## INTRODUCTION

Prostate and colorectal cancers remain among the most common and deadly cancer types worldwide, including Russia. Colorectal cancer is the third most common cancer type worldwide and the second most common cause of cancer-related deaths, whereas prostate cancer is the most common cancer type among men both globally and in Russia [1]. Despite the significant advances in the diagnosis and treatment of these diseases, the high morbidity and mortality rates, particularly at the late stages, necessitate the continuation of studies aimed at identifying new molecular targets for therapeutic intervention. Cancer cell metabolism differs from that of normal cells; however, attempts to use metabolic drugs can be ineffective due to the metabolic plasticity of cancer cells, which use various substrates and alternative survival pathways. One of the new directions in anticancer therapy is the study of the combined action of cytotoxic and metabolic drugs. The idea behind the combination of drugs is to shift the entire metabolism towards only one metabolic pathway under the action of the first component and to inhibit this single pathway using the second component. As a result, the cancer cell gets in a situation when ATP production is impossible or greatly hampered, which leads to its death. Recently, the transcription factor Bach1 (BTB domain and CNC homology 1), which regulates many processes such as aerobic metabolism, heme homeostasis, cell cycle and mitosis, cell aging, and response to oxidative stress [3], has attracted great interest as a new pharmacological target [2]. Increased expression of Bach1 was detected in various types of cancer, including breast cancer [4], lung cancer [5, 6], liver cancer [7], colorectal cancer [8], and prostate cancer [9]. This typically worsens the prognosis for cancer patients, since Bach1 activates invasion and metastasis [10]. Bach1 inhibits mitochondrial metabolism by transcriptionally repressing mitochondrial genes and suppresses pyruvate dehydrogenase activity by activating its kinase expression. Bach1 increases glucose consumption by activating the expression of hexokinase 2 and glyceraldehyde phosphate dehydrogenase. Pharmacological or genetic inhibition of Bach1 can reprogram cells toward increased mitochondrial metabolism and thus increase the sensitivity of cancer cells to inhibition of mitochondrial respiration. This was successfully demonstrated [4] in triple-negative breast cancer cells using a combination of metformin and hemin, a physiological regulator of Bach1 stability. Heme binding in the C-terminal domain of Bach1 leads to its conformational change, release from the nucleus, ubiquitination, and proteasomal degradation [11]. The only well-characterized inhibitor of Bach1 is the compound HPPE, developed by High Point Pharmaceuticals (United States) [12]. Earlier, using comparative transcriptome analysis [13], we showed that it acts similarly to zinc and tin protoporphyrins, known inhibitors of Bach1 [14]. Inhibition of glioblastoma progression in vivo and in vitro using HPPE was recently shown in [15]. Given the fact that metabolic analysis of prostate cancer revealed a distinct contribution of aerobic respiration [16], in this work we for the first time used the Bach1 inhibitors zinc protoporphyrin (ZnPP) and HPPE to assess the effect of combination therapy with the well-known mitochondrial complex I inhibitor rotenone on the viability of prostate and colorectal cancer cell lines.

## MATERIALS AND METHODS

*N*-(2-(2-Hydroxyethoxy)ethyl)-1-methyl-2-((6-(trifluoromethyl)benzo[*d*]thiazol-2-yl)amino)-1H-benzo[*d*]imidazole-5-carboxamide (HPPE) (purity 99.8%) was synthesized to order by Pharmaron (United States). Prostate cancer (PC3 and Du145) and colorectal cancer (HT-29 and HCT-116) cell lines were cultured in DMEM/F12 medium (PanEco, Russia) supplemented with 10% FBS (Capricorn, Germany), 1% Glutamax (Gibco, United States), and 1% Anti-anti (Gibco, United States) at 37°C and 5% CO_2_. Cell growth dynamics were assessed visually using a ZOE Fluorescent Cell Imager inverted microscope (Bio-Rad, United States). To analyze the cell death kinetics, cells were seeded in 96-well plates at a density of 5000 cells per well and incubated in 100 μL of culture medium for 24 h. The culture medium was then replaced with a control medium or a medium containing zinc protoporphyrin, or HPPE, or their combinations with rotenone, and incubated for 48 h. To visualize cell death, the added medium also contained propidium iodide (PI) at a concentration of 1 μg/mL. Cell death kinetics were determined by counting the number of red fluorescent objects (dead cells) over time using the IncuCyte® S3 live cell assay system (Sartorius, United States). The MTT assay was used to analyze the effect of Bach1 inhibitors on cell viability. Briefly, 5000 cells in 100 μL of the nutrient medium were added to each well of a 96-well culture plate and incubated for 24 h in a cell incubator. The nutrient medium was then replaced with a medium containing zinc protoporphyrin, HPPE, and their combinations with respiratory chain inhibitors, and the plate was incubated for another 48 h in a cell incubator. After incubation, 10 μL of an MTT solution in DPBS (5 mg/mL) was added to all wells, and the plate was placed in a cell incubator for 4 h. Then, 100 μL of lysis solution containing 10% SDS, 50% DMF, and acetic acid (pH 4.7) were added to the wells, and the plate was incubated overnight. Thereafter, absorbance was measured at 570 nm using a SpectraMax i3 microplate reader (Molecular Devices, United States). To assess background absorption, wells without cells, to which MTT solution and lysis solution were added, were used.

For real-time PCR, PC3 cells, Du145, HT-29, HCT-116 (5 × 10^4^ cells per well) were seeded in 6-well plates containing 2 mL of complete nutrient solution and incubated at 37°C and 5% CO_2_ for 24 h. The medium was then replaced with a control medium containing 1 μM ZnPP or 10 μM HPPE, after which the cells were incubated for another 24 h. RNA was extracted using the SKYprep RNA Pure Micro Kit (SkyGen, Russia), and its concentration was measured with a NanoDrop ND-1000 spectrophotometer (Thermo Fisher Scientific, United States). Reverse transcription was performed using the MMLV RT kit (Evrogen, Russia). The obtained cDNA was stored at –20°C. PCR was performed using the qPCRmix-HS SYBR dye (Evrogen, Russia), with triplicate runs for each condition. The primer sequence for the reference gene *ACTB*: forward 5'-CTGGAACGGTGAAGGTGACA-3', reverse 5'-AAGGGACTTCCTGTAACAACGCA-3'. The primer sequence for the target gene *HMOX1*: forward 5'-TCAAGCAGCTCTACCGCTCCC-3', reverse 5'-TTGGTGTCATGGGTCAGCAGC-3'. Ct values were obtained at a fluorescence threshold of 5000. Data reliability was assessed by the reproducibility of replicates (Ct spread within one cycle), the absence of product in the negative controls, and the presence of a single peak in the melting temperature curve. PCR analysis was performed using the DTprime system (DNK-Tekhnologiya, Russia).

## RESULTS AND DISCUSSION

**Fig. 1.**
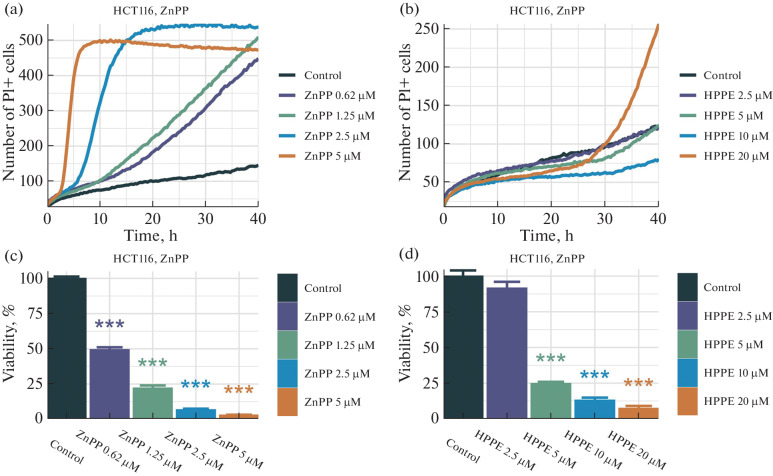
Viability analysis results after treatment with zinc protoporphyrin at concentrations of 5, 2.5, 1.25, and 0.625 μM and with the HPPE agent at concentrations of 20, 10, 5, and 2.5 μM in HCT-116 colorectal cancer cells, assessed using the IncuCyte (top) and MTT (bottom) methods. Significance levels: * (*p* < 0.05), ** (*p* < 0.01), *** (*p* < 0.001).

The study of the kinetics of the separate effects of Bach1 inhibitors on colorectal and prostate cancer cell lines revealed both differences in the effects of ZnPP and HPPE and different sensitivities of the cell lines to the inhibitors. As can be seen from the data for the HCT-116 cell line, presented in Figs. 1a and 1b, ZnPP accelerates cell death and shortens the lag period as its concentration increases, whereas HPPE accelerates cell death but does not shorten the lag period, which is approximately 25–30 h. The same trend holds for the other three cell lines: according to the IncuCyte system data, the HPPE-induced cell death is gradual and slowed. The MTT assay results confirmed these findings. The viability of HCT-116 cells declined exponentially with an IC50 of approximately 0.6 μM for ZnPP (Fig. 1c), at an HPPE concentration of 2.5 μM viability remained close to 100%, whereas an increase in the HPPE concentration to 5 μM decreased viability to approximately 40%.

The fact that zinc protoporphyrin is much more toxic than HPPE was predictable because ZnPP, a non-specific inhibitor of Bach1, can bind to all heme-containing domains of proteins and also inhibit heme oxygenase (HMOX1), an enzyme that is believed to be associated with the ability of cells to survive oxidative stress [14]. HPPE, in addition to inhibiting Bach1, can stabilize Nrf2 and trigger the antioxidant genetic program, thereby increasing the ability of cells to survive [13]. In other words, at short incubation times, HPPE can promote cell survival by activating the antioxidant program. In all lines, mitochondrial respiration makes an insignificant contribution to metabolism, since 1 μM rotenone (which is 2 orders of magnitude higher than the inhibition constant of Complex I) does not have a toxic effect (Table 1); therefore, a concentration of 100 nM was chosen to study its combined effect. Taking into account the sensitivity of cancer lines to the individual effects of ZnPP and HPPE (Table 1), 1 μM ZnPP and 5 μM HPPE were selected for combination with rotenone. The *HMOX1* gene is simultaneously a target of the transcriptional activator Nrf2 and the repressor Bach1; hence, the level of activation of this gene expression is a marker of changes in the Nrf2/Bach1 ratio in the cell under the action of effectors. Therefore, to compare the response of the lines to the action of ZnPP and HPPE, we assessed the activation of the *HMOX1* gene expression for the selected concentrations of Bach1 inhibitors (Table 1).

**Table 1.** Characteristics of the sensitivity of cell lines to the action of individual Bach1 inhibitors

Cell line	Viability, 1 µM rotenone 24 h (MTT test)	Viability, IC50 µM		Induction of HMOX1, times	
		ZnPP	HPPE	ZnPP 1	HPPE 5
Du145	100%	0.30	5.0	1.1	4.6
PC3	100%	0.60	15.0	7.0	27
HCT-116	90%	0.60	5.0	1.6	3.9
HT-29	80%	2.0	15.0	1.9	127

The HT-29 line was the most resistant to the action of ZnPP and HPPE, which may reflect the minimal level of Bach1 in the cell, since the *HMOX1* gene activation under the action of HPPE in this line is many times higher than in other lines (Table 1). The PC3 line apparently has the highest content of Bach1, since the activation of the *HMOX1* gene expression under the action of ZnPP was observed only for this line (Table 1). Lines Du145 and NST-116 showed a similar response to individual action of ZnPP and HPPE, in terms of both viability and activation of *HMOX1* gene expression (Table 1).

**Fig. 2.**
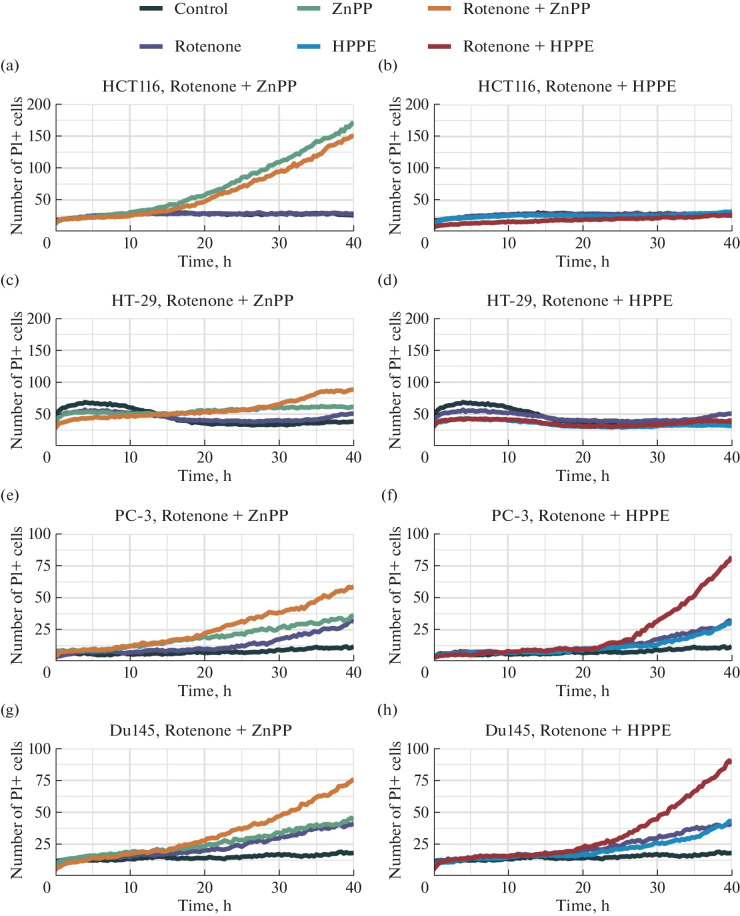
Kinetics of the combined action of 100 nM rotenone and the Bach1 inhibitor (1 μM ZnPP or 5 μM HPPE) on colorectal cancer and prostate cancer cell lines.

Analysis of the kinetics of the combined action of rotenone and the Bach1 inhibitors ZnPP and HPPE showed that the enhancement of the effect of the Bach1 inhibitors in the presence of 100 nM rotenone was observed for both prostate cancer lines, but not for colorectal cancer lines (Fig. 2). The enhancement of the cytotoxic effect in combination with rotenone was most pronounced in the case of HPPE and exceeded the additive enhancement in the presence of ZnPP (Fig. 2). It should be noted that the cytotoxicity of 100 nM rotenone began to manifest itself after 20 h of incubation in the prostate cancer lines, but not in the colorectal cancer lines, in which rotenone did not cause cytotoxicity even after 40 h of incubation. The combination of rotenone and HPPE is nontoxic for both colorectal cancer lines, whereas in the case of ZnPP rotenone does not enhance its toxicity for the HCT-116 line (Fig. 2). The HT-29 line is insensitive to either the individual or combined action of ZnPP or HPPE with rotenone at the selected concentrations (Fig. 2), as could be expected based on the data on the viability of this line in the presence of these inhibitors (Table 1). These results are consistent with the conclusions made in [17], in which an inhibitory effect of *Bach1* gene silencing on migration, but not growth, of HT-29 cells was observed. *Bach1* silencing suppresses migration and invasion in Du145 line [9]. It is also known that, for breast cancer lines, not only migration and invasion are suppressed, but also the cell cycle arrest and growth inhibition are observed [18]. For breast cancer, the effectiveness of combination therapy with metformin and hemin, a Bach1 inhibitor, was also demonstrated [4]. However, it should be noted that in this case we are talking about direct activation of ferroptosis, since hemin is actively processed by heme oxygenase 1 to generate ferrous iron ions, a catalyst of ferroptosis. This was not observed in the case of ZnPP and HPPE, which, on the contrary, inhibit this enzyme.

The differences in the efficacy of combination therapy between colorectal and prostate cancer cell lines, revealed in this study, are apparently due to the greater sensitivity of the latter to rotenone, because during long-term incubation its toxic effect was observed even at 100 nM, suggesting the presence of mitochondrial respiration in prostate cancer cell lines. A possible explanation for the involvement of mitochondria in the metabolism of prostate cancer cell lines is the significant contribution of the metabolic pathway from pyruvate through the Krebs cycle to citrate, followed by lipogenesis [19, 20] and beta-oxidation to produce ATP [21, 22]. Therefore, inhibition of Bach1, which hinders glycolysis, and the simultaneous shutdown of mitochondrial respiration through inhibition of Complex 1 by rotenone, leads to the inability to produce ATP in prostate cancer cells and, thus, to cell death.
